# Bioactive β-Carbolines Harman and Norharman in Sesame Seed Oils in China

**DOI:** 10.3390/molecules27020402

**Published:** 2022-01-09

**Authors:** Wei Liu, Zhaoyu Yang, Lili Shi, Yun Li

**Affiliations:** 1 College of Food Science and Technology, Henan University of Technology, Lianhua Street, Zhengzhou 450001, China; 201892085@stu.haut.edu.cn (Z.Y.); shililislla@163.com (L.S.); 2 Key Laboratory of Agro-Products Safety & Quality of the Ministry of Agriculture, Institute of Quality Standards & Testing Technology for Agro-products, Chinese Academy of Agricultural Sciences, No. 12, Zhongguancun South Street, Beijing 100081, China

**Keywords:** β-carbolines, harman, norharman, sesame seed oil, roasting

## Abstract

The β-carbolines in our diet, mainly including harman and norharman, are a group of biologically active, naturally occurring plant-derived alkaloids. Fragrant sesame seed oil is one of the most popular flavor edible oils in China. Considering that sesame seeds are roasted at 200–240 °C during the processing of flavor sesame seed oils, it is meaningful to investigate the levels of β-carboline compounds in various sesame seed oils. In this work, the levels of β-carbolines (harman and norharman) in different types of sesame seed oils in China (e.g., pressed fragrant sesame oil, ground fragrant sesame oil) have been determined systematically. The results showed that the levels of total β-carbolines in pressed fragrant sesame oils (700.5~2423.2 μg/kg) were higher than that in ground fragrant sesame oils (660.4~1171.7 μg/kg). Roasting sesame seeds at high temperatures (200–240 °C) led to higher levels of β-carbolines (660~2400 μg/kg) in fragrant sesame seed oils. In addition, the loss of tryptophan might be attributed to the formation of β-carbolines in sesame seeds during the roasting process. In general, fragrant sesame seed oils (pressed fragrant sesame oils, ground fragrant sesame oils) contain higher levels of β-carbolines due to the formation of harman and norharman during the roasting sesame seed process.

## 1. Introduction

β-Carbolines, mainly including harman and norharman, are a group of naturally occurring, plant-derived alkaloids that are biologically active in our diets [1,2,3]. Harman (H) and norharman (NH) are also considered as the non-polar heterocyclic aromatic amines (HAAs), generated during the treatment of protein-rich foods (e.g., meat) at high temperature through pyrolysis of proteins and amino acids [4]. Many studies have disclosed a possible correlation between enhanced cancer risk and heterocyclic aromatic amines intake [5,6]. In addition, β-carboline compounds harman and norharman exhibit neuroactive activity in the human body [1,2].

In the past decades, harman and norharman have been detected in many processed and stored foods, including cookies, maize, soy, coffee and even fermented alcoholic beverages [2,7,8,9]. As a popular beverage, coffee, including brewed coffee and roasted coffee beans, has relatively high concentrations of β-carbolines [2]. For example, in coffee grounds and instant coffee, the levels of norharman and harman have been determined to be 0.09–9.34 μg/g and 0.04–1.41 μg/kg, respectively [7]. Many studies have shown that these β-carbolines (harman and norharman) were found in coffee and were inhibitors of monoamines oxidase (MAO), and that coffee consumption has been correlated with a lower incidence of Parkinson’s disease (PD) [1,2]. Therefore, coffee is recommended as a healthy drink in many countries. In fact, cigarette smoke (mainstream and sidestream brands of cigarettes) also contains a high concentration of β-carbolines (8990 ng/cigarette for norharman and 3000 ng/cigarette for harman) [8]. However, cigarette smoke is hard to be recommended.

Edible oils, especially vegetable oils, are one of the most important food ingredients in daily life. The average consumption of edible oil is around 28 kg per year in China. However, less attention has been paid to the levels of harman and norharman in edible vegetable oils. Indeed, edible vegetable oils, including soybean oil, palm oil, canola oil, sunflower oil, rapeseed oil and peanut oil, are produced through chemical refining (e.g., soybean oil) or pressing (e.g., palm oil) [10]. Thus, a very low level of β-carboline compounds (harman and norharman) is detected in these kinds of edible oils [11]. In China, flavor vegetable oils (e.g., sesame oil and peanut oil) are very popular due to their special flavor. For instance, when peanut seeds or sesame seeds are roasted at 140–220 °C for 30–60 min before pressing, some special flavor compounds will be produced [12,13]. Thus, the oil seeds, such as sesame seeds, will generate heterocyclic aromatic amines (e.g., harman and norharman) at heating temperature >100 °C. However, limited reports have been disclosed on the level of β-carboline compounds (harman and norharman) in edible oils, especially in flavor vegetable oils (e.g., sesame seed oils) [14].

Sesame seed oil (so called sesame oil) is one of the most popular flavor oils in China; about 400,000 tons of flavor sesame oil are consumed in China every year. Considering the processing of flavor sesame oil (roasting the sesame seed at 200–240 °C) and bioactivity of β-carbolines (harman and norharman), it is meaningful to investigate the level of β-carboline compounds in sesame oils, including pressed fragrant sesame oil, ground fragrant sesame oil and cold-pressed sesame oil. In this work, the level of β-carbolines (harman and norharman) in different sesame seed oils in China has been investigated, and the effect of different processing methods on the levels of harman and norharman in sesame seed oils has been discussed. The effects of roasting temperature and time on the levels of β-carboline compounds (harman and norharman) in sesame seed oils have been investigated as well.

## 2. Results and Discussion

### 2.1. Analysis of β-Carbolines in Fragrant Sesame Seed Oils

Based on sesame seed oil processing methods, sesame seed oils in China can be divided into four types, including: (1) pressed fragrant sesame seed oil, (2) ground fragrant sesame seed oil, (3) cold-pressed sesame seed oil and (4) refined sesame seed oil [15,16]. These types of sesame seed oils, mainly pressed fragrant sesame oil and ground fragrant sesame oil, have different aroma flavors based on the different processing methods. Therefore, different commercial sesame seed oil samples (16 samples from different brands) were purchased from supermarkets in China.

**Figure 1:**
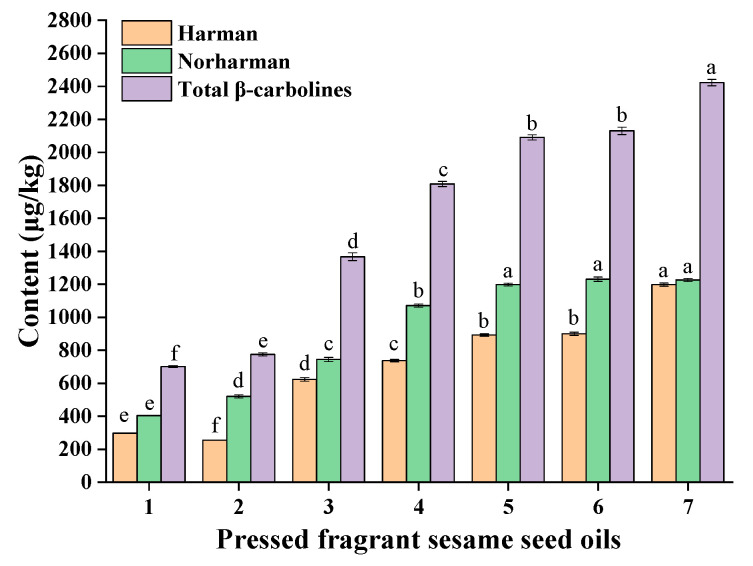
The contents of harman and norharman in pressed fragrant sesame seed oils. Different superscript letters indicate a significant difference at *p*-value < 0.05.

The contents of β-carbolines (harman and norharman) in a batch of different pressed fragrant sesame oils (seven samples) were determined (Figure 1). The results showed that the contents of harman ranged from 254.0 μg/kg to 1197.7 μg/kg and the contents of norharman ranged from 403.6 μg/kg to 1230.7 μg/kg. The total β-carbolines (harman and norharman) ranged from 700.5 μg/kg to 2423.2 μg/kg. Notably, the contents of norharman were higher than the contents of harman in the pressed fragrant sesame oils in most cases.

**Figure 2:**
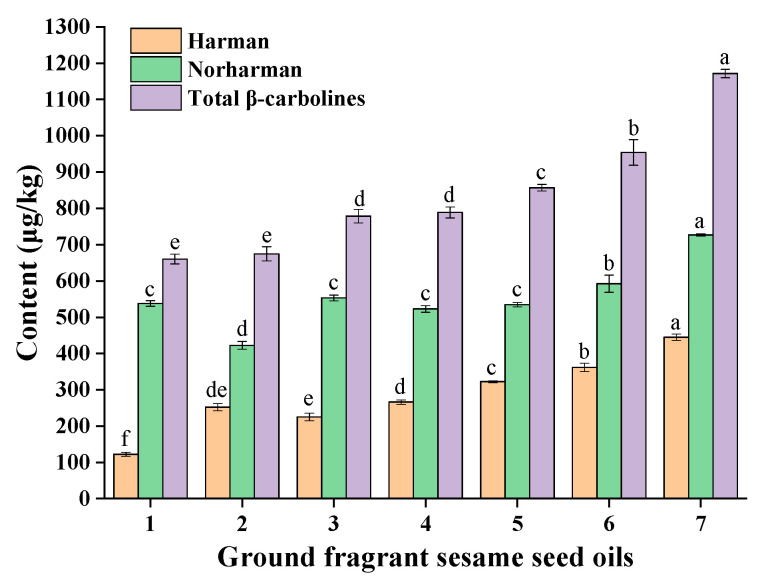
The contents of harman and norharman in ground fragrant sesame seed oils. Different superscript letters indicate a significant difference at *p*-value < 0.05.

The contents of β-carbolines (harman and norharman) in a batch of different ground fragrant sesame seed oils (seven samples) were determined (Figure 2). The results showed that the contents of harman ranged from 122.5 μg/kg to 444.9 μg/kg, the contents of norharman ranged from 422.3 μg/kg to 726.8 μg/kg, and the total β-carbolines (harman and norharman) ranged from 660.4 μg/kg to 1171.7 μg/kg in ground fragrant sesame seed oils. In addition, the contents of norharman were much higher than that of harman in ground fragrant sesame seed oils.

### 2.2. Analysis of β-Carbolines in Cold-Pressed Sesame Seed Oils

Cold-pressed sesame seed oils are produced without sesame seed roasting pretreatment (at higher temperature) before the pressing process. Limited commercial resources could be found in China due to its non-flavor. In fact, cold-pressed sesame seed oils can be used as a good cooking oil for its non-flavor.

**Figure 3:**
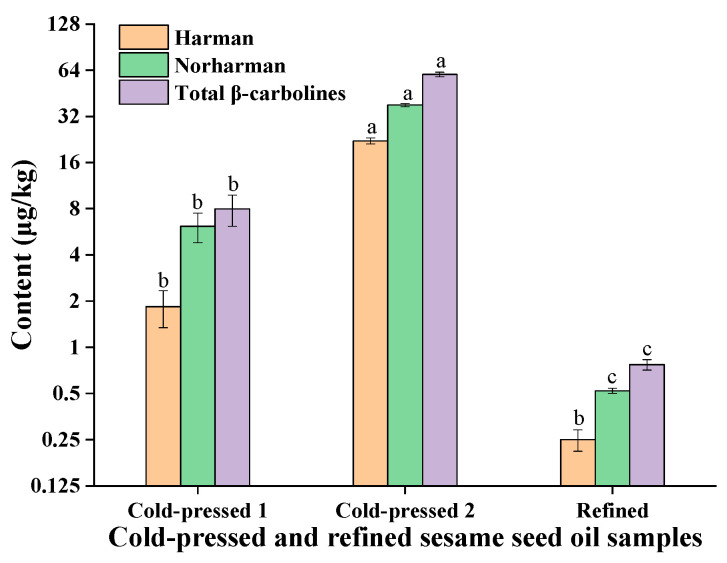
The contents of harman and norharman in cold-pressed and refined sesame oils. Different superscript letters indicate a significant difference at *p*-value < 0.05.

The contents of harman and norharman in two cold-pressed sesame seed oil samples were also determined (Figure 3). The results showed that very low levels of the β-carboline compounds (harman and norharman) were detected, ranging from 8.0 μg/kg to 60.0 μg/kg. The contents of harman ranged from 1.8 μg/kg to 22.1 μg/kg, and the contents of norharman ranged from 6.2 μg/kg to 37.9 μg/kg. Moreover, one refined sesame seed oil sample was analyzed, in which a trace level of the total β-carbolines (harman and norharman) was detected (0.8 μg/kg). Indeed, chemical refining for vegetable oils (e.g., soybean oil) will decrease most of the minor components, such as free fatty acids, vitamin E, phytosterols and other small molecular compounds [17,18,19].

### 2.3. Comparison of Fragrant and Cold-Pressed Sesame Seed Oils

**Figure 4:**
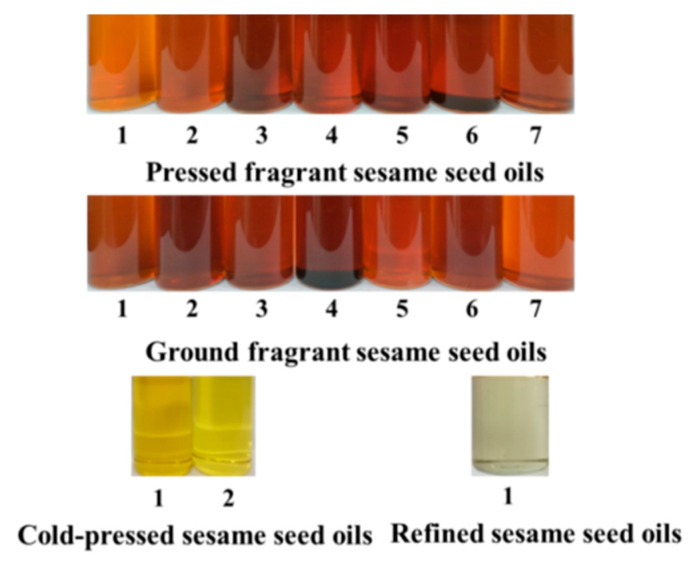
The colors of different sesame seed oil samples in this work.

Based on the above results, it can be concluded that the contents of norharman (6.2 μg/kg–1230.7 μg/kg) were higher than the contents of harman (1.8 μg/kg–1197.7 μg/kg) in all tested sesame seed oil samples (see Figure 1, Figure 2 and Figure 3). The level of total β-carboline compounds (harman and norharman) in sesame seed oil samples followed the order: pressed fragrant sesame seed oil > ground fragrant sesame seed oil > cold-pressed sesame seed oil > refined sesame seed oil. In fact, oil color is an important indicator of oil processing methodologies. It was observed that roasting sesame seeds at higher temperatures led to a brown color of fragrant sesame seed oils (pressed fragrant sesame seed oils and ground fragrant sesame seed oils) compared with cold-pressed sesame seed oils or refined sesame seed oils (Figure 4). Notably, other heterocyclic aromatic amines (HAAs) were not detected in the above sesame seed oil samples (Table 1, Figures S1 and S2).

**Figure 5:**
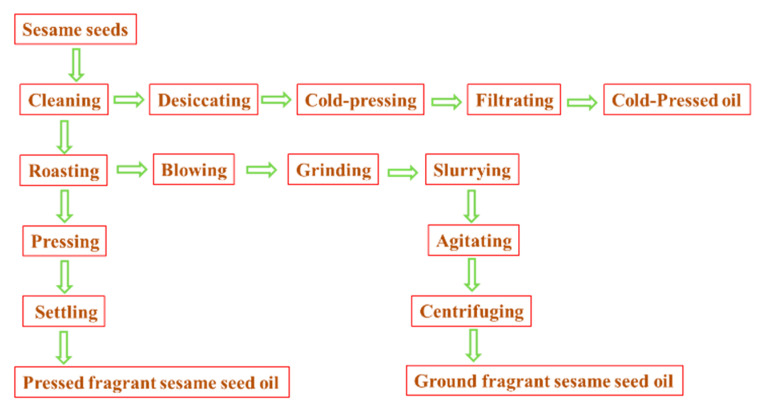
The processing procedure of different sesame seed oils.

Indeed, roasting of sesame seeds at 200–240 °C for 30–60 min is required at the first stage of pressed fragrant sesame seed oil and ground fragrant sesame seed oil processing (Figure 5) [20,21], which will lead to the production of possible heterocyclic aromatic amines (e.g., harman and norharman) due to pyrolysis of amino acids or proteins at higher temperature (like other protein-rich foods). For the processing of ground fragrant sesame seed oil (Figure 5), unit operations slurrying and agitating with hot water (90–95 °C) would remove some β-carboline compounds (harman and norharman) from the sesame seed oil phase [22]. Thus, the processing of traditional Chinese ground fragrant sesame seed oil is called aqueous extraction for sesame seed oil [23,24]. Therefore, it was found that the levels of β-carboline compounds (harman and norharman) in ground fragrant sesame seed oils were lower than the levels of β-carbolines (harman and norharman) in pressed fragrant sesame seed oils (see Figure 1 and Figure 2). Compared with pressed fragrant sesame seed oil and ground fragrant sesame seed oil, cold-pressed sesame seed oil only requires drying the sesame seed at mild heating temperature (<80 °C) before the sesame seed pressing process [16]. Notably, cold-pressed sesame seed oils do not belong to the flavor edible oils (e.g., fragrant sesame seed oils). For refined sesame seed oil, conventional refining, including degumming, deacidification, bleaching and deodorization, will decrease most of the lipid content (e.g., free fatty acids, vitamin E, phytosterols) and small molecular compounds (e.g., nonpolar heterocyclic aromatic amines harman and norharman). Thus, the levels of harman and norharman were as low as 0.8 μg/kg in the refined sesame seed oil sample (see Figure 3).

### 2.4. Effect of Sesame Seed Roasting Process on the Level of β-Carbolines

**Figure 6:**
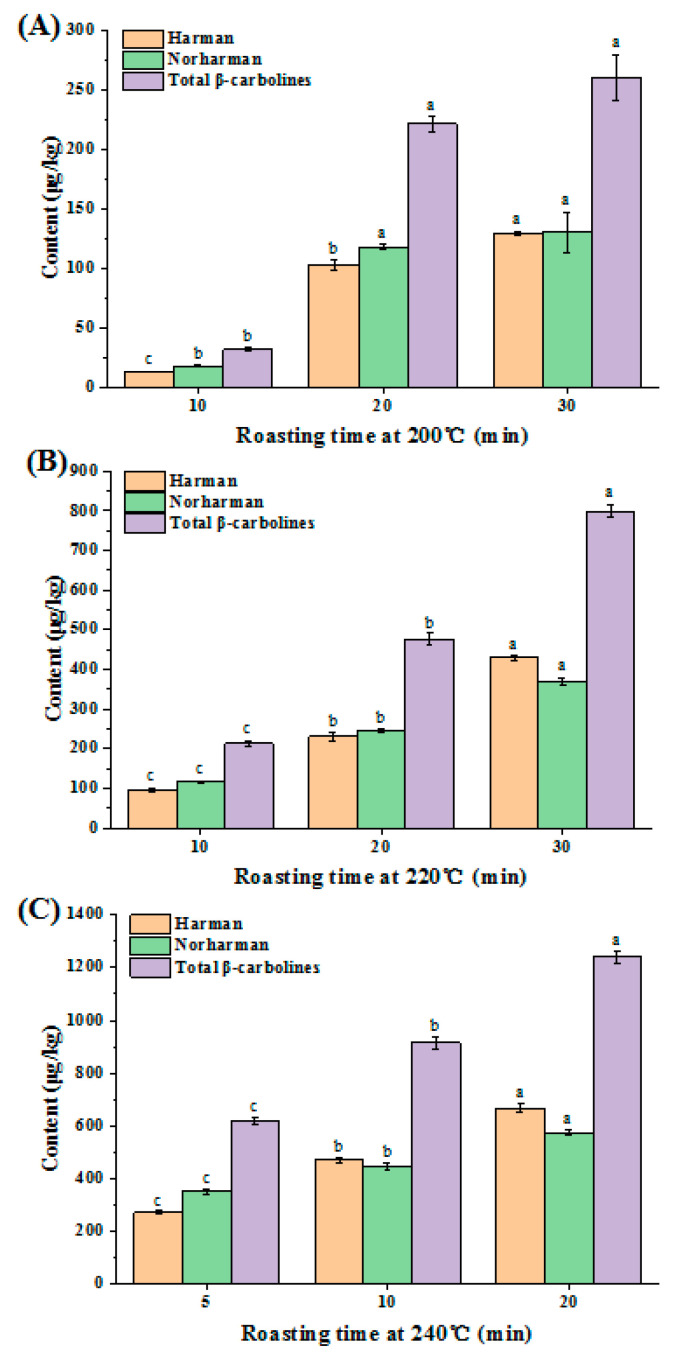
The contents of harman (H) and norharman (NH) of sesame seed oils (SSO) by roasting sesame seeds at 200 °C (**A**), the contents of H and NH of SSO by roasting at 220 °C (**B**), the contents of H and NH of SSO by roasting at 240 °C (**C**). Different superscript letters indicate a significant difference at *p*-value < 0.05.

To investigate the effect of sesame seed roasting temperature on the formation of β-carboline compounds (harman and norharman) in sesame seed oils, a model roasting process was conducted at different roasting temperatures (200 °C, 220 °C, 240 °C), and the results were summarized in Figure 6. The results demonstrated that when roasting sesame seeds at 200 °C, the total β-carboline compounds (harman and norharman) in sesame seed oil increased from 32.2 μg/kg to 260.0 μg/kg with the roasting time prolonging from 10 min to 30 min (Figure 6A). When sesame seeds were roasted at 220 °C, the total β-carboline compounds (harman and norharman) in sesame seed oil increased rapidly from 213.0 μg/kg to 799.8 μg/kg with roasting time increasing from 10 min to 30 min (Figure 6B). By increasing the roasting temperature of sesame seeds to 240 °C, the contents of total β-carboline compounds (harman and norharman) in sesame seed oil rose quickly to 618.4 μg/kg in 5 min, and the contents of harman and norharman reached 1239.7 μg/kg in 20 min (Figure 6C).

In most cases, the levels of norharman in the total β-carbolines was higher than the levels of harman at different roasting temperatures (200–240 °C) (Figure 6). It was concluded that the decreasing level of total β-carboline compounds (harman and norharman) in sesame seed oils roasted at different temperatures followed the order: 240 °C > 220 °C > 200 °C, suggesting that increasing roasting temperature and time indeed led to increasing levels of harman and norharman.

**Figure 7:**
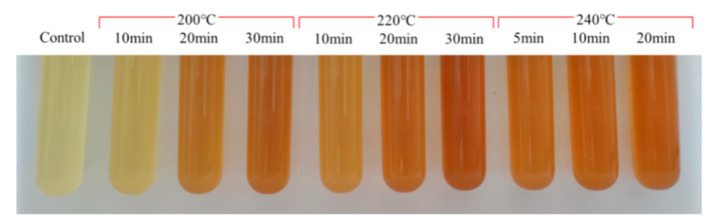
The color of sesame seed oils through roasting sesame seeds at different temperatures.

Moreover, it was observed that the sequence of the oil color of different sesame seed oils after roasting at different temperatures followed the same order: 240 °C > 220 °C > 200 °C, and prolonging of roasting time was attributed to the color-deepening of sesame seed oils (Figure 7). The above results suggested that sesame seed oil color would be a good indicator of the roasting degree of sesame seeds.

In addition, the fatty acid compositions of sesame seed oil at different roasting temperatures (200–240 °C) were determined (Table 2). The sesame seed oil contained palmitic acid (C16:0, 9.68%), stearic acid (C18:0, 6.09%), oleic acid (C18:1, 42.42%) and linoleic acid (C18:2, 41.81%). Indeed, the content of linoleic acid (C18:2), as the main polyunsaturated fatty acid in sesame seed oil, decreased (from 41.81% to 40.96%) when the roasting temperature increased to 220 °C and 240 °C, which mainly led to a decrease in total unsaturated fatty acids (C18:1, C18:2) in sesame seed oils. The loss of linoleic acid (C18:2) under roasting conditions was attributed to the formation of carbonylic compounds, which would promote the formation of β-carboline compounds (harman and norharman) [25].

**Figure 8:**
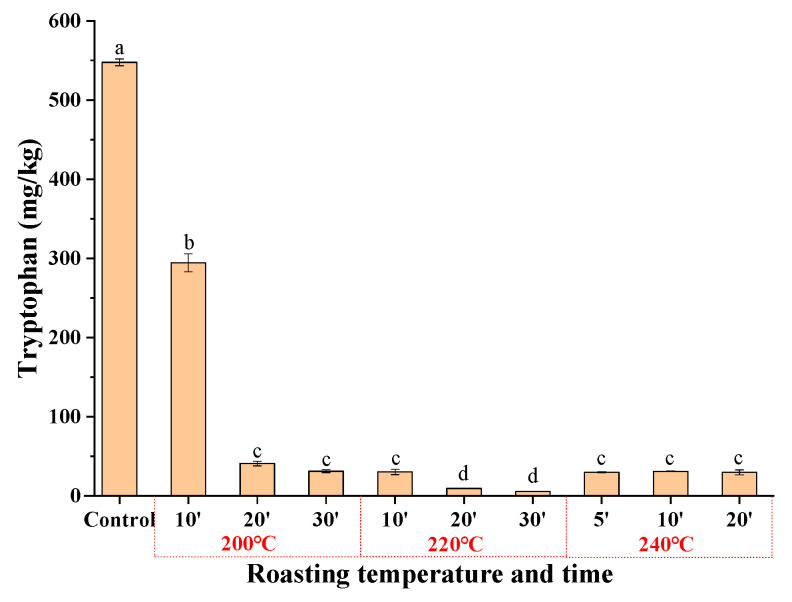
The content of free tryptophan in sesame seeds at different roasting temperatures. Different superscript letters indicate a significant difference at *p*-value < 0.05.

The formation mechanism of β-carboline compounds (harman and norharman) in sesame seed oil was also discussed. In fact, harman and norharman, as non-polar heterocyclic aromatic amines, are usually assigned as pyrolysis products of amino acids at higher temperatures (>150 °C) [26], while tryptophan is considered as the main precursor of β-carboline compounds [9]. Tryptophan may slowly react with released aldehydes, producing tetrahydro-β-carboline-3-carboxylic acid, which is oxidized to give β-carbolines (harman and norharman) [27,28]. Some studies have demonstrated that the total amino acid (detected 17 amino acids) content decreased with increased roasting time at 160–200 °C during the roasting treatment of sesame seeds [29]. However, the change of tryptophan was not included in their study. Therefore, the content of tryptophan was determined when sesame seeds were roasted at 200–240 °C (Figure 8). The results showed that the free tryptophan content decreased rapidly from original 547.6 mg/kg to 294.4 mg/kg after roasting at 200 °C for 10 min, and it reduced rapidly to 31.2 mg/kg with roasting time increasing to 30 min. When roasting sesame seeds at 220 °C, the tryptophan content decreased rapidly to 30.3 mg/kg (roasting for 10 min) and 5.7 mg/kg (roasting for 30 min). At the same time, the contents of other amino acids indeed decreased with increasing roasting time, which was attributed to the formation of complicated Maillard reaction products [4].

## 3. Materials and Methods

### 3.1. Materials

Commercial sesame seed oils, including pressed fragrant sesame seed oils (n = 7) and ground fragrant sesame seed oils (n = 7), were purchased from a local supermarket in China. Sesame seeds were purchased from a local supermarket in China. Acetonitrile (HPLC grade) was purchased from Thermo Fisher Scientific (Shanghai, China). Ammonium hydroxide and hydrochloric acid (HPLC grade) were obtained from Kemiou Chemical Reagent Co. Ltd. (Tianjin, China). Methyl alcohol, acetic acid and n-hexane were of HPLC grade, and other chemicals were of analytical reagent grade. Oasis MCX solid-phase extraction cartridge (150 mg, 6 mL) was purchased from Waters (Milford, PA, USA). The water used was Wahaha purified water purchased from a local supermarket. The standards AαC, MeAαC, DMIP, Trp-P-1, Trp-P-2, Glu-P-2, MeIQ, MeIQx, IQ, PhIP, 4,8-DiMeIQx and 7,8-DiMeIQx were purchased from Toronto Research Chemicals (Toronto, ON, Canada). Harman (1-methyl-9H-pyrido[3,4-b]indole), norharman (9H-pyrido[3,4-b]indole) and 4,7,8-TriMeIQx were purchased from Alta scientific (Tianjin, China).

### 3.2. Methods

#### 3.2.1. Model Roasting Process for Sesame Seeds and Oil Extraction

Sesame seeds (about 1000 g) were roasted using an automatic electric heater (Korea Hanna Co., Seoul, Korea) at different temperatures (200 °C, 220 °C, 240 °C) for the duration of 5 min, 10 min, 20 min and 30 min at each temperature with constant stirring. The roasted sesame seeds (100 g) were crushed with a mortar and then extracted with *n*-hexane three times. The obtained mixture (oil–hexane) was then rotationally vaporized and blown with nitrogen to remove the remaining *n*-hexane. The resulting sesame seed oil was centrifuged to remove impurity particles. All samples were prepared as triplicates.

#### 3.2.2. Analysis of Fatty Acid Composition of Sesame Seed Oil

The fatty acid composition was determined by conversion of oil to fatty acid methyl esters, prepared following the standard IUPAC method 2.301. Fatty acid compositions of sesame seed oils were analyzed by an Agilent Technologies 6890 N gas chromatograph (GC) equipped with a 30.0 m × 250 µm × 0.25 µm BPX-70 capillary column and detected using a flame ionization detector (FID). Samples (1 µL) were injected under the following conditions: the nitrogen flow rate was 1.0 mL/min, the oven was programmed from the set temperature of 170 to 210 °C at 2 °C/min, the split ratio was 50:1, the GC injection temperature was 250 °C, and the detector temperature was 300 °C.

#### 3.2.3. Determination of Free Amino Acids in Defatted Sesame Cake

Free amino acids were analyzed by a Biochrom 30 amino acid analyzer (Biochrom Co. Ltd., Holliston, MA, USA). The extraction and analysis method was according to Song with modification [30]. One gram of defatted sesame cake (accurate to 0.0001 g) was weighed into a 50 mL grinding triangle flask, and 20 mL lithium loading buffer (Biochrom Co. Ltd., USA) was added. Ultrasonic extraction (300 W) was carried out for 30 min. After centrifugation (10,000 rpm) and filtration, the samples were detected by an automatic analyzer.

#### 3.2.4. Extraction, Purification and Analysis of Heterocyclic Aromatic Amines

Heterocyclic aromatic amines (HAAs) were determined by LC-MS based on the literature [31]. Oil samples (1 g) were added with 10μL 5 mg/L 4,7,8-TriMeIQx (internal standard) and combined with 10 mL acetonitrile (containing 1% acetic acid) in a 50 mL centrifuge tube, after which the mixture was shaken for 5 min, followed by ultrasonic extraction for 10 min, and shaken vigorously for 1 min before being centrifuged at 10,000 rpm (−4 °C) for 10 min. The supernatant liquid was collected into a 50 mL centrifuge tube. The above extraction operations with acetonitrile were repeated twice. All extracts were collected together.

A solid phase extraction column cartridge Oasis MCX (150 mg/6 mL) was flushed in advance with 10 mL of methanol and 10 mL 0.1 mol/L HCl–methanol mixed solution (80:20, *v*/*v*). All extracts were transferred to the MCX column for enrichment and purification. Then it was washed with 10 mL water, 10 mL methanol and 10 mL methanol/ammonia/water (25:5:75, *v*/*v*/*v*) mixed solution. Finally, 10 mL methanol/ammonia (95:5, *v*/*v*) solution was used for elution. The eluent was collected and evaporated to dryness under nitrogen. The residue was dissolved in 10 mL 5% formic acid/acetonitrile (95:5, *v*/*v*) mixture and filtered through a 0.45 μm microporous filter for LC/MS analysis.

A Shimadzu LC-20ADXR system coupled with a Triple Quad 3500 mass spectrometer (AB SCIEX, Redwood City, CA, USA) was used to analyze the HAAs of the sample extract. Chromatographic separation was performed on an Agilent ZORBAX Eclipse XDB-C18 column (3.5 μm particle size, 150 mm × 2.1 mm i.d) maintained at 35 °C. The gradient elution was achieved with a binary mobile phase of 5% formic acid/5 mM ammonium formate aqueous solution (A) and 5% formic acid/5mM ammonium formate methanol solution (B) at a flow rate of 0.4 mL/min. The gradient elution program was as follows: 0–0.01 min, 5%B; 0.01–1.00 min, 5%B; 1.00–1.10 min, 5–60%B; 1.10–5.00 min, 60–80%B; 5.00–6.00 min, 80–95%B; 6.00–8.00 min, 95%B; 8.00–8.10 min, 95–5%B; 8.10–8.20 min, 5%; 8.20–10.00 min adjust mobile phase balance to initial state. The single injection volume was set at 5 μL.

MS analysis was carried out with positive electrospray ionization (ESI+). Multiple reaction monitoring (MRM) conditions were automatically optimized. The capillary voltage was 5.5 kV, and the ion source temperature was 550 °C. The MRM parameters for 14 HAAs and internal standard are summarized in Table S1 (see Supplementary Materials).

#### 3.2.5. Statistical Analysis

All experiments were carried out in triplicate, and the mean and standard deviation (SD) for each of the determinations were calculated and reported. Figure preparation was performed using Origin Pro software (Origin Lab Co., Northampton, MA, USA). The differences between groups were tested by ANOVA and Duncan’s multiple range tests. Means were compared, and they were considered significant when *p* < 0.05.

## 4. Conclusions

In this work, the levels of β-carbolines (harman and norharman) in different types of sesame seed oils in China, including pressed fragrant sesame seed oil, ground fragrant sesame seed oil, refined sesame seed oil and cold-pressed sesame seed oil, were investigated systematically. The results showed that the levels of the total β-carbolines (harman and norharman) in pressed fragrant sesame seed oils were higher than those in ground fragrant sesame seed oils, and they were much higher than those in cold-pressed or refined sesame seed oils. Model roasting sesame seeds at higher temperatures (200–240 °C) for 5–30 min led to higher levels of β-carbolines harman and norharman (660~2400 μg/kg) in fragrant sesame seed oils. The loss of tryptophan might be attributed to the formation of β-carbolines (harman and norharman) in sesame seeds during the roasting process at higher temperature. This study will be meaningful to prove a correlation between the contents of β-carbolines in fragrant sesame seed oils and the roasting processes of sesame seeds, and whether fragrant sesame seed oils will be a dietary supplement of β-carbolines (harman and norharman).

## Figures and Tables

**Table 1:** The contents of 14 HAAs were determined in different sesame seed oils (μg/kg) ^a^.

HAA (μg/kg)	Pressed Fragrant Sesame Seed Oils (7 Samples)	Ground Fragrant Sesame Seed Oils (7 Samples)	Cold-Pressed Sesame Seed Oils (2 Samples)
AαC	ND ^b^	ND	ND
MeAαC	ND	ND	ND
Trp-P-1	ND	ND	ND
DMIP	ND	ND	ND
Glu-P-2	ND	ND	ND
MeIQ	ND	ND	ND
MeIQx	ND	ND	ND
IQ	ND	ND	ND
PhIP	ND	ND	ND
4,8-DiMeIQx	ND	ND	ND
7,8-DiMeIQx	ND	ND	ND
Harman	254.0–1197.7	122.5–444.9	1.8–22.1
Norharman	403.6–1230.7	422.3–726.8	6.2–37.9
Trp-P-2	ND	ND	ND

^a^ Not detected (ND); ^b^ Note: heterocyclic aromatic amines (HAAs) were detected by LC-MS.

**Table 2:** Fatty acid compositions (%) of sesame seed oils by roasting sesame seeds at different temperatures.

Fatty Acid	Control	200 °C			220 °C			240 °C		
		10 min	20 min	30 min	10 min	20 min	30 min	5 min	10 min	20 min
C16:0	9.68 ± 0.03 ^d,e^	9.66 ± 0.02 ^e^	9.73 ± 0.09 ^c,d,e^	9.74 ± 0.03 ^c,d^	9.74 ± 0.02 ^c,d^	9.79 ± 0.02 ^b,c^	9.91 ± 0.02 ^a^	9.85 ± 0.00 ^a,b^	9.88 ± 0.01 ^a^	9.92 ± 0.03 ^a^
C18:0	6.09 ± 0.01 ^b^	6.23 ± 0.03 ^a^	6.22 ± 0.10 ^a^	6.26 ± 0.05 ^a^	6.18 ± 0.03 ^a,b^	6.21 ± 0.04 ^a^	6.23 ± 0.01 ^a^	6.19 ± 0.03 ^a,b^	6.20 ± 0.02 ^a^	6.24 ± 0.01 ^a^
ΣSFA	15.77 ± 0.04 ^d^	15.89 ± 0.05 ^c,d^	15.95 ± 0.19 ^b,c^	16.00 ± 0.08 ^a,b,c^	15.93 ± 0.05 ^b,c,d^	15.99 ± 0.06 ^a,b,c^	16.14 ± 0.03 ^a^	16.03 ± 0.03 ^a,b,c^	16.08 ± 0.03 ^ab^	16.16 ± 0.04 ^a^
C18:1	42.42 ± 0.06 ^b^	42.60 ± 0.01 ^b^	42.50 ± 0.13 ^b^	42.58 ± 0.02 ^b^	42.79 ± 0.17 ^a^	42.84 ± 0.09 ^a^	42.88 ± 0.10 ^a^	42.84 ± 0.02 ^a^	42.90 ± 0.07 ^a^	42.88 ± 0.04 ^a^
C18:2	41.81 ± 0.02 ^a^	41.52 ± 0.02 ^b^	41.55 ± 0.06 ^b^	41.42 ± 0.02 ^b,c^	41.28 ± 0.16 ^c,d^	41.17 ± 0.03 ^d,e^	40.99 ± 0.14 ^f^	41.13 ± 0.02 ^d,e,f^	41.02 ± 0.04 ^e,f^	40.96 ± 0.02 ^f^
ΣUFA	84.23 ± 0.08 ^a^	84.11 ± 0.03 ^a,b^	84.05 ± 0.19 ^b,c^	84.00 ± 0.04 ^b,c,d,e^	84.07 ± 0.34 ^b,c^	84.01 ± 0.12 ^b,c,d^	83.87 ± 0.24 ^d,e^	83.97 ± 0.03 ^b,c,d,e^	83.92 ± 0.10 ^c,d,e^	83.84 ± 0.05 ^e^

Note: SFA and UFA are the abbreviations of saturated fatty acids and unsaturated fatty acids, respectively. Different superscript letters indicate a significant difference at *p*-value < 0.05.

## Data Availability

Not applicable.

## Supplementary Materials

The following supporting information can be downloaded online. The supplementary data contain the related MRM parameters for HAAs and internal standard (Table S1), MRM chromatogram and extracted ion chromatograms (EIC) of HAAs (Figures S1 and S2).

## Sample Availability

Samples are available from Wei Liu on valid request.
