# Supplementary material for: Bioactive β-Carbolines Harman and Norharman in Sesame Seed Oils in China
Source: Molecules. 2022 Jan 9;27(2):402. doi: 10.3390/molecules27020402 (PMC8780151; doi:10.3390/molecules27020402)
Supplement: Supplementary file 1 [file molecules-27-00402-s001.zip › molecules-1513665-supplementary.pdf]

Supplementary Materials

Bioactive β-carbolines harman and norharman in sesame seed oils in China

Wei Liu,[1,*] Zhaoyu Yang,[1] Lili Shi,[1] Yun Li[2,*]

[1]College of Food Science and Technology, Henan University of Technology, Lianhua Street, Zhengzhou 450001, P. R. China.

[2]Key Laboratory of Agro-products Safety & Quality of the Ministry of Agriculture, Institute of Quality Standards & Testing Technology for Agro-products, Chinese Academy of Agricultural Sciences, No.12, Zhongguancun South Street, Beijing 100081, China.

***Corresponding author.** Tel/Fax: 086-371-67758022

E-mail address:

liuwei307@hotmail.com (W. Liu)

liyun01@caas.cn (Y. Li)

Table S1. The MRM parameters for 14 HAAs and internal standard (4,7,8-TriMeIQx)

HAAs	Precursor ion[M + H]$^{+}$(m/z)	Diagnostic Productions (m/z)	Cone voltage (V)	Collision voltage (eV)	Dwell time (msecs)
AαC	184.0	167.2	108	32	30
		140.0	108	32	
MeAαC	198.2	154.1	104	40	30
		127.1	104	45	
Trp-P-1	212.0	168.0	80	30	30
		195.2	80	40	
DMIP	162.9	147.3	90	45	30
		105.0	90	45	
Glu-P-2	185.2	131.1	80	40	30
		78.2	80	40	
MeIQ	213.1	198.0	100	35	30
		144.0	100	60	
MeIQx	214.1	199.0	100	40	30
		131.0	100	55	
IQ	199.1	184.0	100	40	30
		157.0	100	50	
PhIP	225.3	210.2	120	45	30
		183.2	120	50	
4,8-DiMeIQx	228.1	211.8	100	45	30
		160.0	100	40	
7,8-DiMeIQx	228.0	131.3	100	55	30
		213.2	100	40	
4,7,8-DiMeIQx	242.0	227.1	120	40	30
		145.0	120	50	
Harman	183.0	115.0	120	50	30
		168.3	120	40	
Norharman	169.2	115.0	100	45	30
		142.0	100	40	
Trp-P-2	198.0	154.0	60	40	30
		128.0	60	40	

Note: heterocyclic aromatic amines (HAAs) were detected by LC-MS.

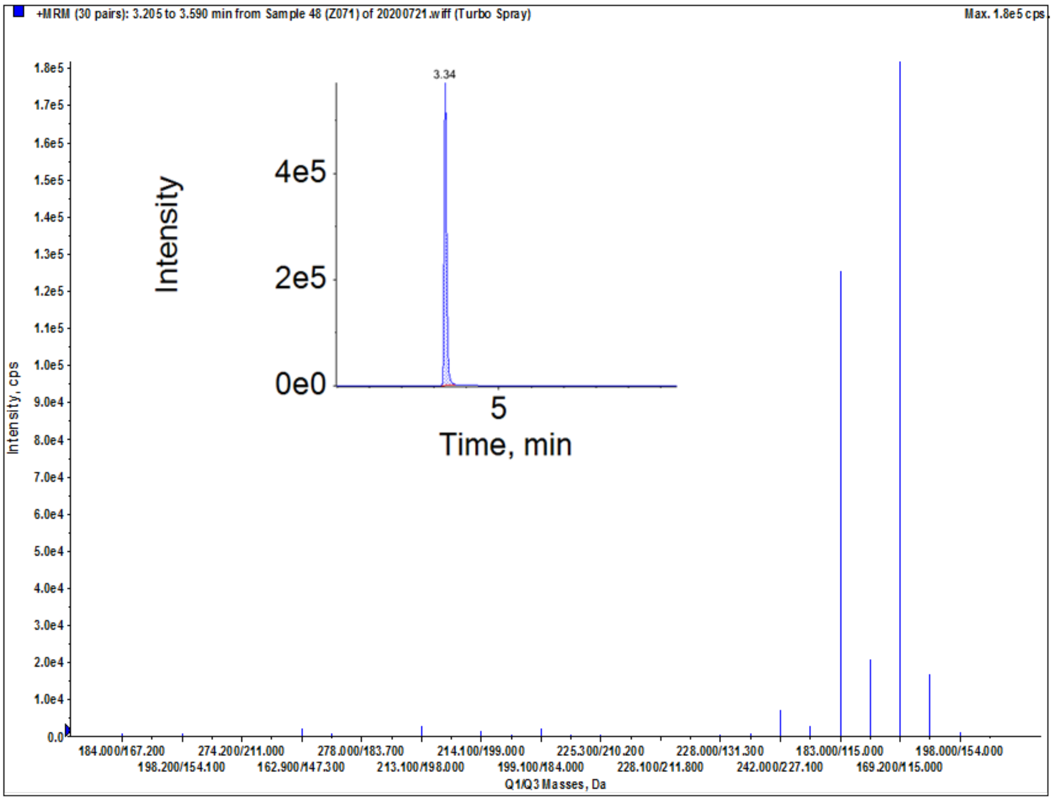

Figure S1. MRM chromatogram and extracted ion chromatograms (EIC) of harman in sesame seed oil sample

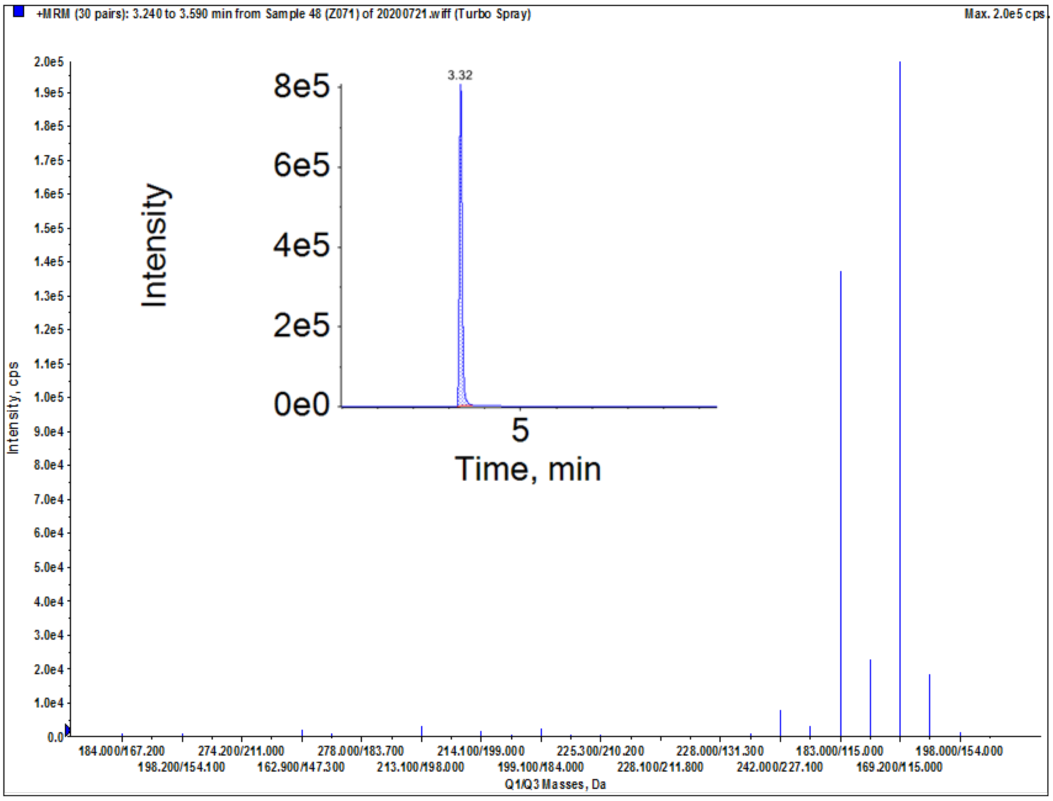

Figure S2. MRM chromatogram and extracted ion chromatograms (EIC) of norharman in sesame seed oil sample
